# PRL1, an RNA-Binding Protein, Positively Regulates the Accumulation of miRNAs and siRNAs in Arabidopsis

**DOI:** 10.1371/journal.pgen.1004841

**Published:** 2014-12-04

**Authors:** Shuxin Zhang, Yuhui Liu, Bin Yu

**Affiliations:** 1 Center for Plant Science Innovation & School of Biological Sciences, University of Nebraska-Lincoln, Lincoln, Nebraska, United States of America; 2 Biotechnology Research Institute, Chinese Academy of Agricultural Sciences & Key Laboratory of Agricultural Genomics, Ministry of Agriculture, Beijing, China; The University of North Carolina at Chapel Hill, United States of America

## Abstract

The evolutionary conserved WD-40 protein PRL1 plays important roles in immunity and development. Here we show that PRL1 is required for the accumulation of microRNAs (miRNAs) and small interfering RNAs (siRNAs). PRL1 positively influences the processing of miRNA primary transcripts (pri-miRNAs) and double-stranded RNAs (dsRNAs). Furthermore, PRL1 interacts with the pri-miRNA processor, DCL1, and the dsRNA processors (DCL3 and DCL4). These results suggest that PRL1 may function as a general factor to promote the production of miRNAs and siRNAs. We also show that PRL1 is an RNA-binding protein and associates with pri-miRNAs in vivo. In addition, *prl1* reduces pri-miRNA levels without affecting pri-miRNA transcription. These results suggest that PRL1 may stabilize pri-miRNAs and function as a co-factor to enhance DCL1 activity. We further reveal the genetic interaction of PRL1 with CDC5, which interacts with PRL1 and regulates transcription and processing of pri-miRNAs. Both miRNA and pri-miRNA levels are lower in *cdc5 prl1* than those in either *cdc5* or *prl1*. However, the processing efficiency of pri-miRNAs in *cdc5 prl1* is similar to that in *cdc5* and slightly lower than that in *prl1*. Based on these results, we propose that CDC5 and PRL1 cooperatively regulate pri-miRNA levels, which results in their synergistic effects on miRNA accumulation, while they function together as a complex to enhance DCL1 activity.

## Author Summary

PRL1, a conserved WD-40 protein, is required for plant development and immune responses. However, its functional mechanisms are not well understood. Here, we show the positive impact of PRL1 on the accumulation of miRNAs and siRNAs, which are key regulators of plant growth and immunity. PRL1 interacts with multiple DCLs (the processors of miRNAs and siRNAs) and is required for their optimal activities, suggesting that PRL1 acts as a general factor to facilitate the production of miRNAs and siRNAs. In addition, PRL1 is an RNA-binding protein, binds pri-miRNAs in vivo and positively influences the levels of pri-miRNAs levels without affecting the promoter activities of genes encoding pri-miRNAs. These results suggest that PRL1 may also stabilize pri-miRNAs. We further show that RPL1 and its interactor CDC5 (a DNA-binding protein) synergistically regulate pri-miRNA levels, resulting in enhanced effects on miRNA accumulation, although they function together as a complex to facilitate DCL1 activity.

## Introduction

In plants and animals, microRNAs (miRNAs), ∼20–25 nucleotides (nt) in size, regulate gene expression in various biological processes such as development and metabolism [1]–[3]. They are produced as duplexes through precise excision from imperfect fold-back primary transcripts (pri-miRNAs) [1]–[3]. In the miRNA duplex, the miRNA strand is loaded into ARGONAUTE (AGO) proteins to repress the expression of target genes containing its complementary sequences while the other strand (passenger strand; miRNA*) is degraded [1]–[3]. Plants and animals also use small interfering RNAs (siRNAs) to repress gene expression. siRNAs are chemically identical to miRNAs [2]. However they are produced from long double stranded RNAs. The two major classes of plant siRNAs are siRNAs derived from repeated DNAs (ra-siRNAs) and trans-acting siRNAs (ta-siRNAs) [4], [5].

In plants, most pri-miRNAs are transcribed by DNA-dependent RNA polymerase II (Pol II) from endogenous miRNA encoding genes (*MIR*) [1], [2]. The mediator complex and Negative on TATA less2 (NOT2; a transcription factor) regulate the transcription of *MIR* [6], [7]. After generation, pri-miRNAs are proposed to be stabilized by DAWDLE (DDL), an RNA binding protein [8]. Pri-miRNAs are then processed to stem-loop precursors (pre-miRNAs) and finally to the miRNA/miRNA* duplex by Dicer-LIKE 1 (DCL1; an RNAse III enzyme) in the nucleus in plants [9], [10]. The C2H2 zinc-finger protein SERRATE (SE) and the RNA binding proteins HYPONASTIC LEAVES 1 (HYL1) and TOUGH (TGH) form a complex with DCL1 to ensure efficient and accurate process of pri-miRNAs [9], [11]–[17]. To ensure its proper function, HYL1 needs to be dephosphorylated during pri-miRNA processing [18]. Several other proteins including DDL, Cap-Binding Protein 20 (CBP20), CBP80, RACK1 and NOT2 are associated with the DCL1 complex to facilitate miRNA maturation [7], [8], [19]–[21]. NOT2 and MODIFIER OF SNC1, 2 (MOS2; an RNA binding protein) have been shown to guide the correct localization of the DCL1 complex [7], [22]. SICKLE (SIC; a proline rich protein) is shown to regulate the accumulation of some miRNAs [23]. Besides protein factors, the structure of pri-miRNAs plays essential roles in regulating DCL1 activity [24]–[27]. For instance, an imperfectly paired lower stem of ∼15 bp below the miRNA:miRNA* duplex is crucial for the initial pri-miRNA cleavage [25]–[27].

We previously showed that Cell Division Cycle 5 (CDC5), a DNA-binding protein, positively regulates miRNA biogenesis in Arabidopsis [28]. CDC5 interacts with Pol II and *MIR* promoters [28]. Lack of CDC5 reduces the occupancy of Pol II at *MIR* promoters and pri-miRNA levels, suggesting that CDC5 is a positive transcription factor of *MIR* [28]. Besides acting as a transcription factor, CDC5 functions as a co-factor of the DCL1 complex to participate pri-miRNA processing [28]. CDC5 is a component of the conserved MOS4-associated complex (MAC). MAC was first identified as a suppressor of *snc1*, which carries a gain-of-function mutation in the *SNC* gene and show constitutive resistance to a wide spectrum of pathogens [29]. Loss-of-function mutations in the MAC complex reduce plant immunity to bacterial infections and cause multiple developmental defects such as reduced fertility and delayed growth [29]. The counterparts of MAC in yeast and Human associate with spliceosome and function in splicing [29]. Other components of MAC include MOS4 (a coil-coil domain containing protein), PRL1 (a WD-40 protein), MAC3A and MAC3B (two functionally redundant U-box E3 ubiquitin ligases). Among these proteins, PRL1 and MOS4 have been shown to interact with CDC5 directly [29].

In this study, we show that PRL1 but not MOS4 plays important roles in the accumulation of miRNAs and siRNAs. Lack of PRL1 in *prl1* reduces miRNA accumulation and pri-miRNA processing efficiency. In addition, PRL1 interacts with the DCL1 complex, suggesting it may function as co-factor of DCL1 to promote miRNA maturation. Pri-miRNA levels are reduced *in prl1* relative to wild-type plants. However, *MIR* promoter activity is not affected by *prl1*, despite of the association of PRL1 with Pol II. Based on the facts that PRL1 is an RNA-binding protein and binds pri-miRNAs *in vivo*, we propose that PRL1 may stabilize pri-miRNAs. Furthermore, the levels of both miRNAs and pri-miRNAs are further reduced in *cdc5 prl1* relative to either *cdc5* or *prl1*. However, CDC5 and PRL1 do not show additive effects on the processing of pri-miRNAs. These results suggest that CDC5 and PRL1 may synergistically influence pri-miRNAs levels and act together as a complex to promote miRNA maturation. PRL1 also interacts with DCL3 and DCL4, which produces siRNAs, and is required for their optimal activities, suggesting that PRL1 may be a general accessory factor for the production of small RNAs.

## Results

### The accumulation of miRNAs and siRNAs is reduced in *prl1-2*

**Figure 1:**
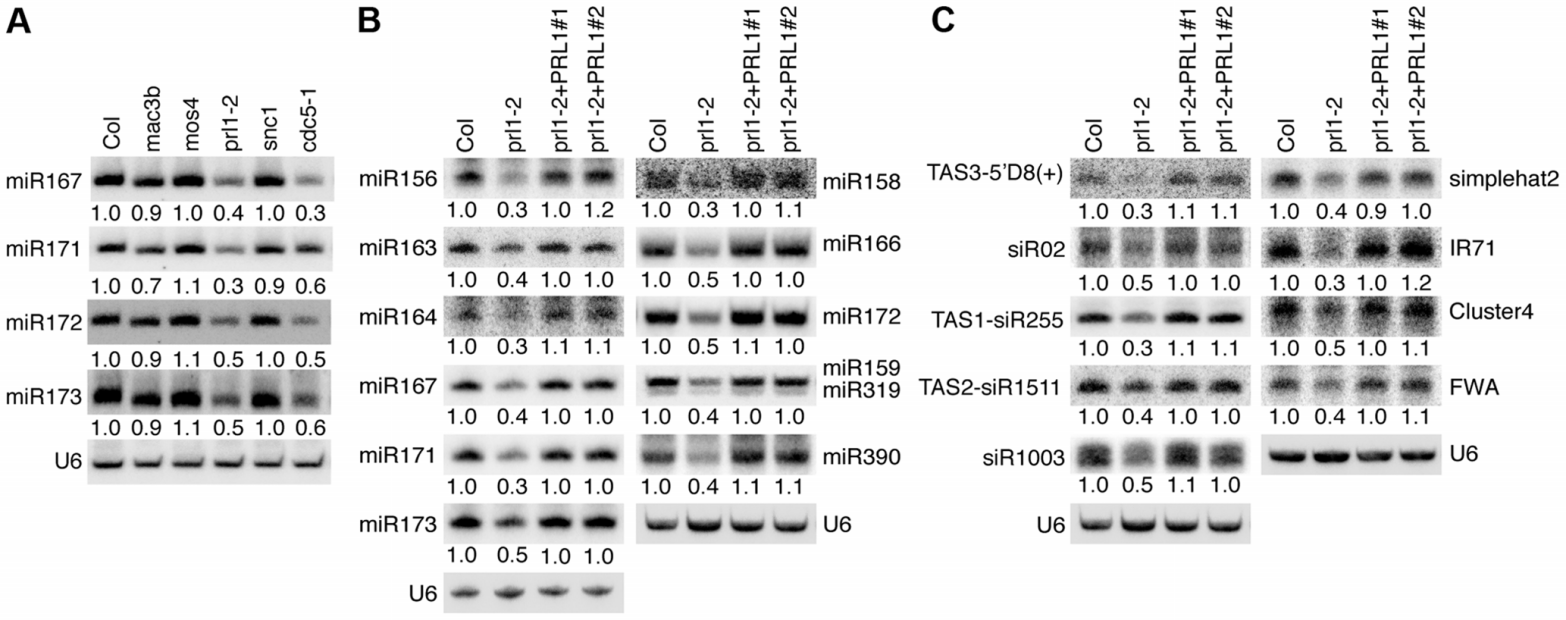
PRL1 is required for the accumulation of miRNAs. (A) The effect of various MAC components on the abundance of miRNAs (B) The levels of miRNAs are reduced in *prl1-2*. (C) The levels of siRNAs are reduced in *prl1-2*. Col: wild-type control. For miR159/319: upper band, miR159; lower band, miR319. Northern blot was used to detect small RNAs and the radioactive signals were quantified with ImageQuant (V5.2). To determine the amount of miRNAs/siRNAs in various mutants relative to that in Col, the radioactive signals of miRNAs/siRNAs were normalized to U6 RNA and compared with that in Col (set as 1). The numbers indicate the average value of three repeats (P<0.05).

Given the role of CDC5 in miRNA biogenesis, it is possible that other components of the MAC complex may also be required for miRNA accumulation. Therefore, we examined the effect of the mutants *mac3b* (SALK_050811), *mos4* (SALK_0090851C) and *prl1-2* on miRNA abundance using Northern blot. We also included *snc1* (SALK_047058C) in the analysis since SNC1 is related to the MAC complex and *snc1* causes development defects. These mutants are likely null since the transcripts of corresponding genes could not be detected by RT-PCR (Figure S1A). Like in *cdc5-1*, the abundance of all four tested miRNAs (miR167, miR171, miR172 and miR173) was decreased in *prl1-2* compared to Col (wild-type control). In contrast, miRNA levels in *mos4*, *mac3b* and *snc1* were comparable with those in Col (Fig. 1A). We examined the accumulation of additional miRNAs in *prl1-2* and found that all these miRNAs were reduced in abundance in *prl1-2* relative to Col (Fig. 1B). In addition, expression a wild-type copy of *PRL1* fused with a *YFP* tag under the control of its native promoter (*pPRL1::PRL1-YFP*) fully recovered miRNA levels in *prl1-2* (Fig. 1B). These results demonstrated that PRL1 but not MOS4 and MAC3b is required for miRNA accumulation. We next analyzed the transcript levels of several miRNA targets (*ARF3*, *CUC1*, *MYB33*, *MYB65* and *PHV*) in *prl1-2* and Col by quantitative RT-PCR (qRT-PCR) in order to test the effect of *prl1-2* on miRNA function. The transcription levels of these targets were slightly increased in *prl1-2* relative to Col (Figure. S1B). The PRL1 transgene fully recovered miRNA function in *prl1* (Figure S1B). We also asked if PRL1 has a role in siRNA biogenesis. The levels of nine examined siRNAs (three ta-siRNAs and six ra-siRNAs) were reduced compared to those in Col (Fig. 1B and 1C), which was complemented by the expression of *pPRL1::PRL1-YFP*. These results revealed that like CDC5, PRL1 positively regulates the levels of miRNAs and siRNAs in Arabidopsis.

### PRL1 associates with Pol II and DCL1

**Figure 2:**
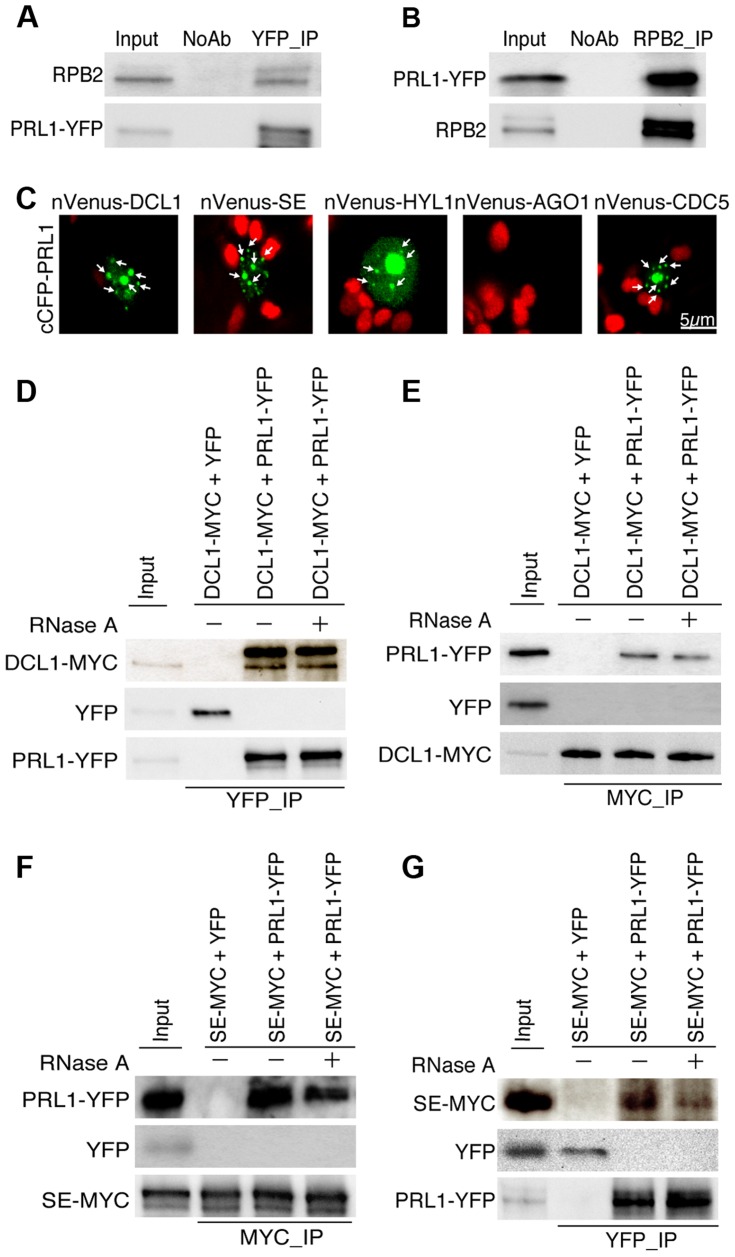
PRL1 associates with the Pol II and DCL1 complexes. (A) and (B) Co-immunoprecipitation (Co-IP) between PRL1 and Pol II. Protein extracts from transgenic plants containing PRL1-YFP were incubated with Anti-YFP or anti-RPB2 antibodies to precipitate PRL1-YFP or Pol II. PRL1-YFP and RBP2 were detected with western blot and labeled on the left side of the picture. Ten percent of input proteins were used for IP and one percent of input proteins were used for Co-IP. (C) BiFC analysis of PRL1 with DCL1, HYL1, SE, AGO1 and CDC5. Paired cCFP- and nVenus-fusion proteins were co-infiltrated into *N. benthamiana* leaves. The BiFC signal (Yellow fluorescence) was detected at 48 h after infiltration by confocal microscopy, assigned as green color and marked with arrow. 30 nuclei were examined for each pair and an image is shown. Red: auto fluorescence of chlorophyll. (D) and (E) Co-immunoprecipitation between PRL1 and DCL1. (F) and (G) Co-immunoprecipitation between PRL1 and SE. PRL1-YFP or YFP were co-expressed with DCL1-MYC and SE-MYC in *N. benthamiana*, respectively. Anti-YFP and anti-MYC (MBL) antibodies were used to detect YFP- and MYC-fused proteins, respectively. The protein pairs in the protein extracts were indicated on the on tope of the picture and proteins detected by western blot were indicated on the left side of the picture. Ten percent of input proteins were used for IP and one percent of inputs proteins were used for Co-IP.

The PRL1-CDC5 interaction suggests that similar to CDC5, PRL1 may act as a co-factor of Pol II and DCL1 to regulate miRNA accumulation. To test these two possibilities, we first examined the PRL1-Pol II association using co-immunoprecipitation (co-IP) assay. In this experiment, anti-YFP and anti-RPB2 that detects the second largest subunit of Pol II (RPB2) [6] were used to capture the PRL1-YFP and Pol II complex, respectively, from the protein extracts of *prl1-2* complementation line expressing the *pPRL1::PRL1-YFP* transgene. After IP, PRL1-YFP was detected in the Pol II precipitates whereas RPB2 existed in the PRL1-YFP complex (Fig. 2A and 2B). In contrast, no interaction was detected in the control reactions (Fig. 2A and 2B), demonstrating the PRL1-Pol II association.

We next tested the association of PRL1 with the components of DCL1 complex using a bimolecular fluorescence complementation (BiFC). In the BiFC assay, transient co-expression of PRL1 fused with C-terminal fragment of cyan fluorescent protein (cCFP) with DCL1, SE, HYL1 or CDC5 fused with the N-terminal fragment of Venus (nVenus) produced yellow fluorescence signals (Fig. 2C), suggesting that PRL1 might associate with the DCL1 complex. To verify this result, we tested co-IP of PRL1 with DCL1 and SE. After PRL1-YFP or YFP was transiently co-expressed with DCL1-MYC and SE-MYC fusion proteins in *N. benthamiana*, respectively, IPs were performed with anti-YFP or anti-MYC antibodies. Western blots detected PRL1-YFP in the DCL1-MYC and SE-MYC complexes and DCL1-MYC/SE-MYC in the PRL1-YFP precipitates, suggesting that PRL1-YFP and DCL1/SE reciprocally pulled down each other (Fig. 2D, 2E, 2F and 2G). As a control, YFP did not show interaction with either DCL1 or SE. These results demonstrated the association of PRL1 with DCL1 and SE.

### PRL1 positively influences pri-miRNA levels without affecting *MIR* promoter activity

**Figure 3:**
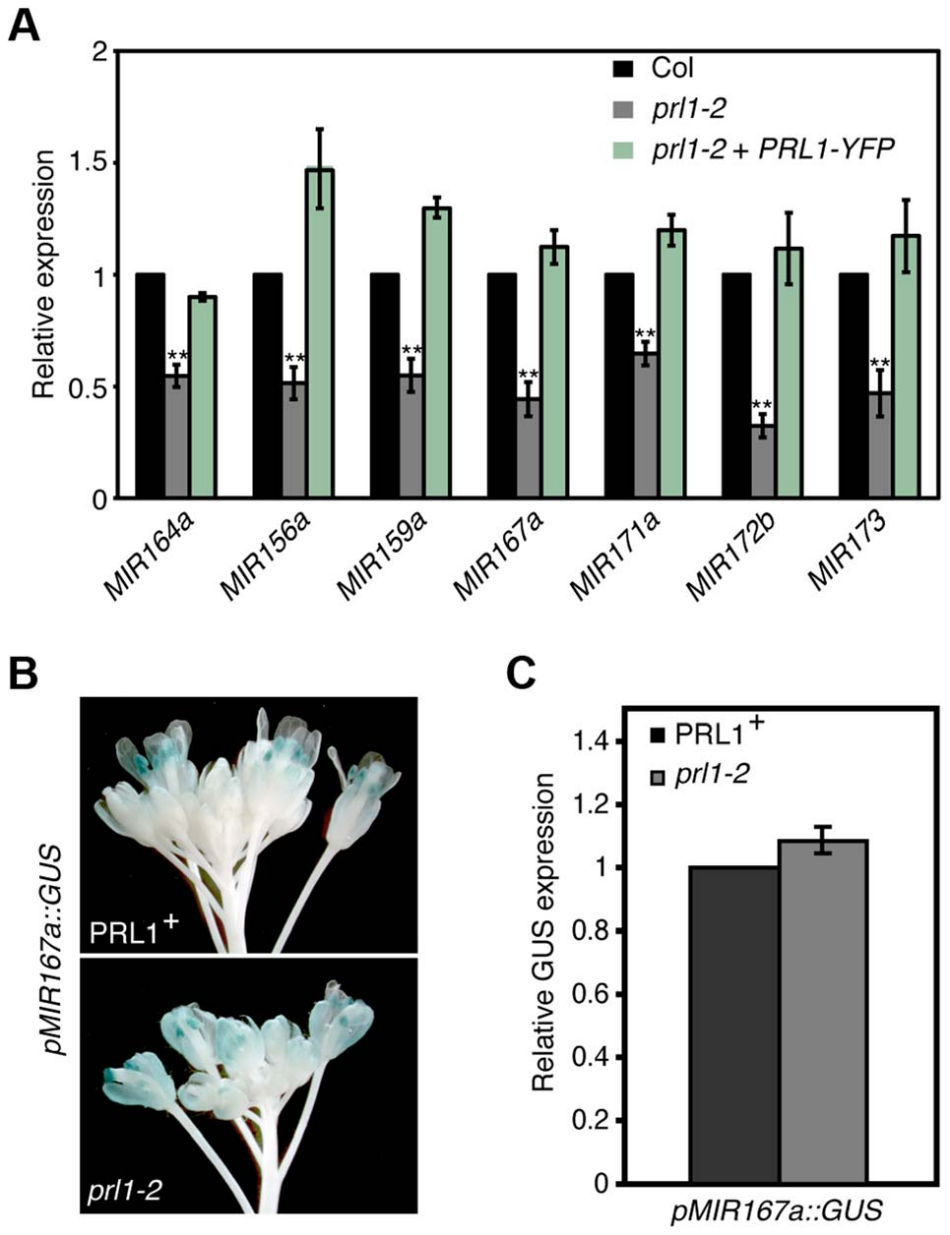
PRL1 positively influences pri-miRNA levels. (A) The abundance of pri-miRNAs in inflorescences of *prl1-2* and Col. The transcript levels of pri-miRNAs in *prl1-2* were determined by quantitative RT-PCR (qRT-PCR), normalized to that of *UBQUITIN5* (*UBQ5*) and compared with those in Col. Value of Col was set to 1. Error bars indicate standard deviation of three technical replications. **: P<0.01. (B) GUS expression in *PRL1^+^* and *prl1-2* harboring *pMIR167a::GUS*. *PRL1^+^*: *PRL1/PRL1* or *PRL1/prl1-2*. Twenty plants containing GUS were stained for each genotype and an image was shown. (C) Relative *GUS* mRNA levels in *PRL1^+^* and *prl1-2* harboring *pMIR167a*::GUS. *GUS* transcript levels were determined by qRT-PCR and normalized to *UBQ5*. Value of *PRL1^+^* was set to 1. Standard deviation of three technical replications was shown as error bars. P = 0.11 (t-test).

The interaction of PRL1 with Pol II suggests that PRL1 may positively regulate *MIR* transcription. If so, lack of PRL1 will impair *MIR* transcription, resulting in reduced levels of pri-miRNAs. To test this, we compared the pri-miRNA levels in *prl1-2* with those in Col using qRT-PCR. In fact, the levels of all seven examined pri-miRNAs in *prl1-2* were less than those in Col (Fig. 3A), which were recovered in the complementation line of *prl1-2* (Fig. 3A). To test whether the reduction of pri-miRNA levels is due to impaired *MIR* promoter activity, we introduced the *prl1-2* mutation into a Col transgenic line containing a single cope of *GUS* transgene driven by *MIR167a* promoter (*pMIR167a::GUS*), which was previously used to test the function of the mediator complex in regulating *MIR* transcription [6]. However, GUS staining and qRT-PCR analysis showed a similar *GUS* expression level in *PRL1^+^* (*PRL1/PRL1* or *PRL1/prl1* genotype) and *prl1-2* containing the *pMIR167a::GUS* transgene (Fig. 3B and 3C). This result demonstrated that PRL1 does not affect *MIR* promoter activity. Consistent with this notion, *prl1* did not show obvious effect on *MIR172b* promoter activity (Figure S2A).

We next evaluated the effect of *prl1-2* on the half-lives of pri-miRNAs using cordycepin as a transcriptional inhibitor [30]. Two-week old plants were transferred to medium containing cordycepin. After incubation was stopped at various time points, we measured the levels of pri-miR164a and pri-miR167a using qRT-PCR. The results showed that the degradation rate of pri-miR164a and pri-miR167a in *prl1-2* is similar to that in Col (Figure S2B).

### PRL1 functions in miRNA maturation

**Figure 4:**
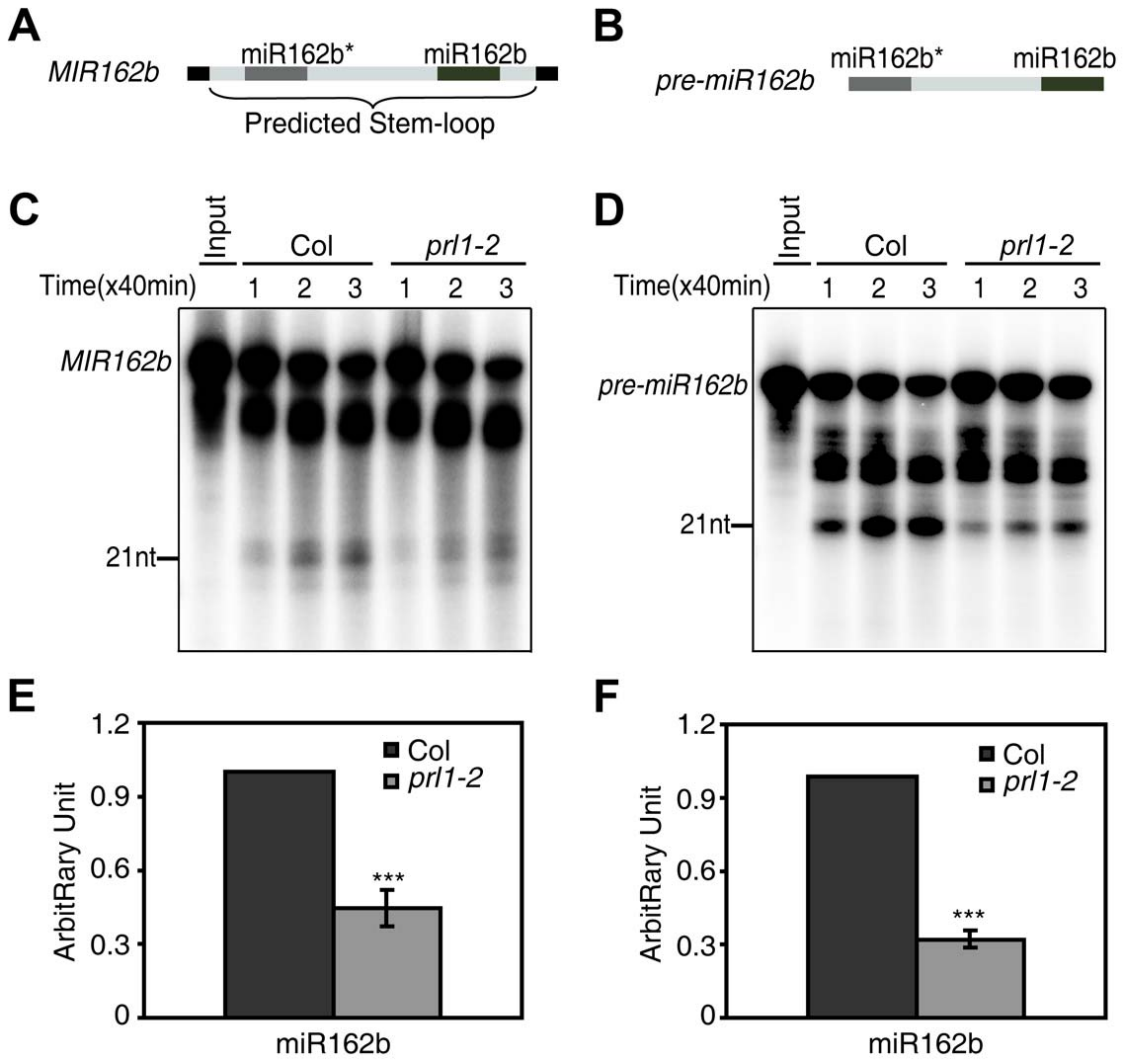
PRL1 is required for miRNA maturation *in vitro*. (A) and (B) A schematic diagram of the *MIR162b* (A) and *pre-miR162b* (B) used *in vitro* processing assay. (C) and (D) The amount of miR162b produced from *MIR162b* and *pre-miR162b* were reduced in *prl1-2*. Proteins were isolated from inflorescences of *prl1-2* and Col and incubated with *MIR162b* or *pre-miR162b*. The reactions were stopped at various time points as indicated in the picture. (E) and (F) Quantification of miR162b production in *prl1-2* compared to that in Col. Quantification analysis was performed at 80 min. The radioactive signal of miR162 were normalized to input and compared with that of Col. The amount of miR162 produced in Col was set as 1. The value represents mean of three repeats (*** *P*<0.001; t-test).

We next asked whether PRL1 has a role in processing of miRNA precursors through an *in vitro* processing assay [13], [31] since it is associated with DCL1 and SE. In this experiment, a portion of *pri-miR162b* that contains the stem-loop of miR162b with 6-nt arms at each end (*MIR162b*; Fig. 4A) and *pre-miR162b* (Fig. 4B) were first produced through *in vitro* transcription in the presence of [α-^32^P] UTP. [^32^P]-labeled *MIR162b* and *pre-miR162b* were then processed in the protein extracts of young flower buds of *prl1-2* or Col. The production of miR162b from both *MIR162b* and *pre-miR162b* in *prl1-2* at various time points was less than that in Col (Fig. 4C and 4D). The processing of MIR162b and pre-miRNA162b was recovered in the PRL1 complementation line (Figure S3) The levels of miR162 produced from *MIR162b* and *pre-miRNA162b* in *prl1* at 80 min were ∼40% of those produced in Col (Fig. 4E and 4F). These results suggested that PRL1 might have a role in promoting miRNA maturation.

### The role of PRL1 in siRNA biognesis

**Figure 5:**
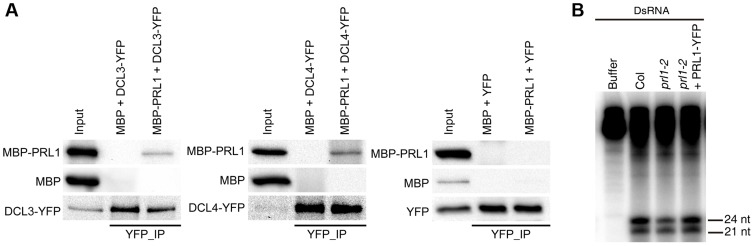
The role of PRL1 in siRNA biogenesis. (A) PRL1 interacts with DCL3 and DCL4. Co-IP was performed to detect the interaction of PRL1 with DCL3 or DCL4. MBP and MBP-PRL1 fused protein were expressed in *E.coli*. YFP, DCL3-YFP and DCL4-YFP were expressed in *N. benthamiana* leaves. Anti-YFP was used for IP. For loading, ten percent and one percent of input proteins were used for IP and Co-IP, respectively. (B) *prl1-2* impairs siRNA production from double-stranded RNAs (dsRNAs). Protein extracts isolated from inflorescences of Col, *prl1-2* and *prl1-2* containing a PRL1-YFP transgene were incubated dsRNAs for 120 min. dsRNAs were synthesized through *in vitro* transcription of a DNA fragment (5′ portion of *UBQ5* gene, ∼460 bp) under the presence of [α-^32^P] UTP.

We next asked the role of PRL1 in siRNA biogenesis, as *prl1-2* reduces the accumulation of siRNAs. By analog, we examined the interaction of PRL1 with DCL3 and DCL4 and the effect of *prl1-2* on dsRNA processing. To test the PRL1-DCL3/DCL4 interaction, we expressed a recombined PRL1 fused with a maltose-binding protein at its N-terminus (MBP-PRL1) and MBP in *E.coli*. The protein extracts containing MBP-PRL1 or MBP were mixed with protein extracts containing DCL3-YFP or DCL4-YFP, which were transiently expressed in *N. benthamiana*. Then the DCL3-YFP or DCL4-YFP complex was IPed with anti-YFP antibodies. MBP-PRL1, but not MBP, was co-IPed with DCL3-YFP and DCL4-YFP (Fig. 5A). In addition, YFP did not interact with MBP or MBP-PRL1. These results demonstrated that PRL1 interacts with DCL3 and DCL4 (Fig. 5A).

To test the effect of *prl1* on dsRNA processing, we generated ∼460 bp dsRNAs through in vitro transcription of a DNA fragment (5′ portion of *UBIQUITIN 5*) containing the T7 promoter at the end of each strand under the presence [α-^32^P] UTP. The radioactive labeled dsRNA then incubated with *prl1* or Col protein extracts. The production of both 21 nt and 24 nt small RNAs was impaired in *prl1* compared with that in Col and that in the complementation line (Fig. 5B). This result indicated that multiple DCL activities are impaired by *prl1-2*, because DCL3 is responsible for the production of 24 nt small RNAs and DCL1/DCL4 is involved in the production of 21 nt small RNAs from dsRNAs.

### PRL1 and CDC5 synergistically regulate miRNA accumulation

**Figure 6:**
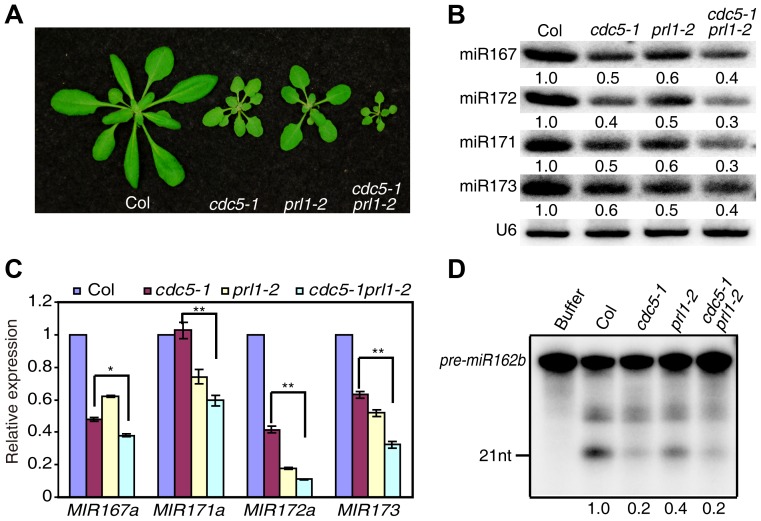
PRL1 and CDC5 synergistically regulate miRNA accumulation. (A) Morphological phenotypes of Col, *cdc5-1*, *prl1-2* and *cdc5-1 prl1-2*. (B) The abundance of miRNAs is lower in *cdc5-1 prl1-2* than that in *cdc5-1* or *prl1-2*. Small RNAs were detected by Northern Blot. To determine the amount of miRNAs, radioactive signals of miRNAs were normalized to U6 RNA. The number represents the relative abundance compared to Col (set as 1) quantified by three repeats (P<0.05). (C) The abundance of pri-miRNAs is reduced in *cdc5-1 prl1-2*. The levels of pri-miRNAs in various mutants were determined by qRT-PCR, normalized to *UBQUITIN5* (*UBQ5*) and compared with those of Col (set as 1). Standard deviation of three technical replications was shown as error bars. **: P<0.01. (D) miR162b production from *pre-miR162b* in Col, *cdc5-1 prl1-2*, *cdc5-1* and *prl1-2*. The reaction was stopped at 120 min. The radioactive signals of miR162b were normalized to input. The number represents the relative production in various genotypes compared to Col (set as 1) quantified by three repeats (P<0.05).

CDC5 and PRL1 have been shown to directly interact with each other. Both CDC5 and PRL1 interact with DCL1 and positively regulate miRNA processing. These results raise a possibility that CDC5 and PRL1 may act as a complex to regulate DCL1 activity. In addition, CDC5 regulates MIR promoter activity while PRL1 does not. These suggest that PRL1 and CDC5 might act additionally in miRNA pathway. To test these two possibilities, we constructed a *cdc5-1 prl1-2* double mutant by crossing *prl1-2* into *cdc5-1* and compared miRNA levels in *cdc5-1 prl1-2* with those in *cdc5-1* and *prl1-2*, respectively. The *cdc5-1 prl1-2* double mutant displayed more severe developmental defects than either *cdc5-1* or *prl1-2*, suggesting that PRL1 and CDC5 function additionally in regulating development (Fig. 6A). Northern blot analyses showed that the levels of several examined miRNAs in *cdc5-1 prl1-2* were lower than those in either *prl1-2* or *cdc5-1* (Fig. 6B), indicating that PRL1 and CDC5 function synergistically in miRNA pathway.

There are at least two possible explanations for the further reduction of miRNA levels in *cdc5-1 pr1l-2* relative to either *cdc5-1* or *prl1-2* based on the fact that both PRL1 and CDC5 positively regulate pri-miRNA levels and miRNA maturation. One is that pri-miRNA levels might be further reduced in *cdc5-1 prl1-2*. The other is that the processing efficiency of miRNA precursors might be lower than either *cdc5-1* or *prl1-2*. To test these two possibilities, we first determined the pri-miRNA levels in *cdc5-1 prl1-2*, *cdc5-1* and *prl1-2* through qRT-PCR. The levels of several pri-miRNAs were decreased in *cdc5-1 prl1-2* when compared with those in either *cdc5-1* or *prl1-2* (Fig. 6C), demonstrating that CDC5 and PRL1 indeed act synergistically in regulating pri-miRNA levels. Next, we evaluated the *in vitro* processing of pre-miR162b in *cdc5-1 prl1-2*. The amount of miR162b produced in *cdc5-1 prl1-2* was similar to that in *cdc5-1* and slightly lower than that in *prl1-2* (Fig. 6D), suggesting that PRL1 and CDC5 may not act additionally in promoting miRNA maturation.

### PRL1 binds pri-miRNAs *in vitro* and *in vivo*

**Figure 7:**
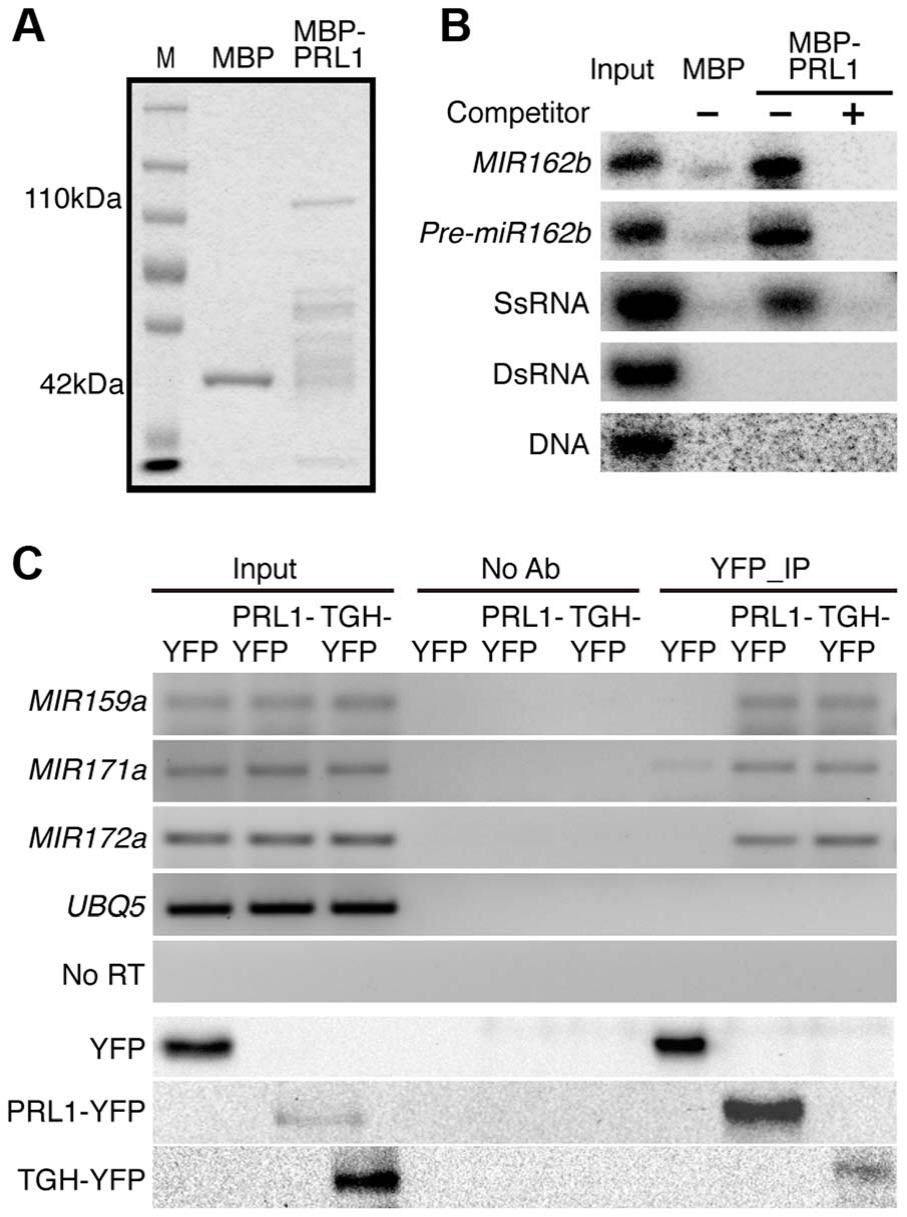
PRL1 binds pri-miRNAs *in vitro* and *in vivo*. (A) Proteins used for *in vitro* RNA binding assay. Purified MBP and MBP-PRL1 proteins were resolved on SDS-page gel and stained by Coomassie Blue. The lower bands in MBP-PRL1 line are degraded MBP-PRL1 since they can be detected by anti-MBP antibody. M: marker. (B) PRL1 binds *MIR162b*, *pre-miR162b* and ssRNA *in vitro*. *MIR162b*, *pre-miR162b* and ssRNA were produced by *in vitro* transcription. SsRNA: single stranded-RNA; DsRNA: double stranded-RNA. (C) PRL1 binds pri-miRNAs *in vivo*. NoAb: No antibody control. Transgenic plants harboring *pPRL1::PRL1-YFP*, *pTGH:* TGH-YFP or *YFP* transgene were used for RIP assay with anti-YFP anti-bodies. Ten percent immunoprecipitates and two percent input proteins were analyzed by western blot. Five percent RNAs were used as input RNA.

Given the role of PRL1 in RNA metabolism, it is reasonable to speculate that PRL1 might have an RNA-binding activity. We therefore performed an *in vitro* RNA pull-down assay to test this possibility. In this assay, recombinant PRL1 fused with a maltose-binding protein at its N-terminus (MBP-PRL1) and MBP were expressed in *E.coli* and purified with amylose resin (Fig. 7A). MBP-PRL1 and MBP were then incubated with [^32^P]-labeled *MIR162b or pre-miR162b*, respectively. MBP-PRL1 but not MBP bound *MIR162b* and *pre-miR162b* after incubation. In addition, when excess amount of unlabeled *MIR162b* or *pre-miR162b* was added in the reaction, radioactive labeled *MIR162b or pre-miR162b* could not be retained in the MBP-PRL1 complex. These results suggested that PRL1 binds RNAs *in vitro*. We next tested the binding ability of MBP-PRL1 to dsRNA, ssRNA and DNA using the in vitro RNA pull-down assay described above. MBP-PRL1 was able to bind a ∼100-nt RNA corresponding to a portion of the 5′ end of the *UBIQUITIN 5* (*UBQ5*) [13], but not an in vitro synthesized ∼50 bp DNA fragment [32] and a dsRNA generated through in vitro transcription vitro transcription of a DNA fragment (5′ portion of *UBQ5*, ∼460 bp) containing the T7 promoter at end of each strand [13] (Fig. 7B).

Next, we performed an RNA immunoprecipitation (RIP) assay to test whether PRL1 binds pri-miRNAs *in vivo* [13]. Seedlings of the *prl1-2* complementation line expressing *pPRL1::PRL1-YFP* transgene and the control plants harboring *YFP* were used for RIP. After PRL1-YFP or YFP complex were precipitated with anti-YFP antibody, pri-miRNAs were detected with RT-PCR. Several tested pri-miRNAs (pri-miR159a, pri-miR167a, pri-miR171 and pri-miR172a) existed in the PRL1-YFP complex but not in the YFP complex and no Anti-body (NoAb) controls (Fig. 7C). These results suggested that PRL1 associates with pri-miRNAs *in vivo*.

## Discussion

In this study, we identify PRL1, a WD-40 protein, as an important regulator of miRNA accumulation. Several evidences including reduced accumulation of pri-miRNAs and miRNAs in *prl1*, PRL1-DCL1 interaction and PRL1-pri-miRNA association demonstrate that PRL1 positively impacts miRNA biogenesis. It has been suggested that PRL1 influences plant immunity and development through its impacts on RNA processing [29], [33]. Given the essential roles of miRNAs in plant immunity and development, it is possible that reduced miRNA levels in *prl1* may partially contribute to the observed phenotypes.

PRL1 likely has a role in promoting miRNA maturation, as lack of PRL1 reduces processing of *MIR162b* and *pre-miR162b*. PRL1 interacts with the DCL1 complex and does not positively regulate the transcription of genes involved in miRNA biogenesis (Figure S4), suggesting that PRL1 may act as a co-factor to regulate DCL1 activity. CDC5, a direct interactor of PRL1 also regulates the DCL1 activity through its interaction with the helicase and dsRNA binding domains of DCL1. The effect of PRL1 on pri-miRNA processing appears to be weaker than that of CDC5. The processing efficiency of *MIR162b* and *pre-miR162b* in *cdc5-1 prl1*-*2* is similar to that in *cdc5-1* and slightly lower than that in *prl1-2*. This result suggests that PRL1 and CDC5 may act together as a complex to regulate DCL1 activity. Furthermore, gel filtration analysis suggests that PRL1 may not affect DCL1-CDC5 association (Figure S5). Thus, it is possible that PRL1 may act as accessory factor to facilitate CDC5 function.

PRL1 also positively regulates the pri-miRNA levels since *prl1* reduces the accumulation of pri-miRNAs. We previously showed that CDC5 interacts with Pol II and positively regulate *MIR* transcription [28]. Since PRL1 associates with Pol II as well, it is possible that PRL1 acts as a component of the CDC5 complex to regulate *MIR* promoter activity. However this seems not to be the case, as loss-of-function of PRL1 does not affect the *GUS* levels driven by the *MIR167a* promoter. Consistent with this notion, the levels of pri-miRNAs are further reduced in *cdc5-1 prl1-2* compared with *cdc5-1* or *prl1-2*. Given the fact that PRL1 binds pri-miRNAs in *vitro* and *vivo*, we propose that PRL1 may stabilize pri-miRNAs. Indeed, the fact that the half-life of pri-miR164a and pri-miR167a in *prl1* is similar to that in Col suggests that the degradation of pri-miRNAs may be increased in *prl1*, because less efficient processing may lead to increased abundance of pri-miRNAs in *prl1*. However, we cannot rule out the possibility that PRL1 acts in *MIR* transcription after initiation, as it associates with Pol II.

In summary, we reveal that PRL1 positively regulates miRNA levels through its impacts on pri-miRNA levels and processing. PRL1 functions additively with its interactor CDC5 as miRNA abundance is lower in *cdc5-1 prl1-2* than in *cdc5-1* or *prl1-2*. The synergistic effect of CDC5 and PRL1 on miRNA levels can be explained by their different roles in controlling pri-miRNA levels rather than their function in promoting miRNA maturation. Besides CDC5 and PRL1, the core components MAC complex includes MOS4, MAC3A and MAC3B [29]. We show that MOS4 and MAC3b have no impact on miRNA levels. However, whether MAC3B has a role in miRNA accumulation needs to be further explored since it acts redundantly with MAC3A [29]. The MAC complex appears to have a role in siRNA biogenesis. Both CDC5 and PRL1 promote the accumulation of siRNA [28] while MOS4 is required for the accumulation of ra-siRNAs [34]. How does MAC participate in siRNA biogenesis? We have showed both PRL1 and CDC5 interact with the DCL1 complex and regulate its activity. By analogy, it is possible that the MAC complex associates with the DCL3 complex to regulate its activity. In fact, DCL3 interacts with PRL1. *prl1* also reduces the abundance of ta-siRNAs, whose production requires DCL4 and DCL1-dependent miRNAs. Since PRL1 interacts with DCL4 and is required for the accumulation of DCL1-dependent miRNAs, it may promote ta-siRNA production through facilitating DCL4 function and miRNA production. The MAC complex is an evolutionarily conserved complex [29]. As many aspects of small RNA pathway are conserved, it is tempting to propose that the counterparts of MAC play some roles in small RNA pathways in other organisms.

## Materials and Methods

### Plant materials

The *mac3b* (SALK_050811), *mos4* (SALK_0090851C), *prl1-2* (Salk_008466), *snc1* (SALK_047058C) and *cdc5-1* (SAIL_207_F03) mutants were ordered from *Arabidopsis* Biological Resources Center (ABRC). All of them are in the Columbia-0 genetic background. Transgenic line containing a single copy of *pMIR167a::GUS* was crossed to *prl1-2*. In the F2 population, *PRL1/PRL1*, *PRL1/prl1-2* and *prl1-2/prl1-2* harboring *pMIR167a::GUS* were identified through PCR genotyping for *prl1-2* and *GU*S.

### Plasmid construction

*PRL1* genomic DNA was amplified from Col genome and cloned to pMDC204 binary vector to generate *pPRL1::PRL1-YFP* construct. The construct was transformed to *prl1-2*. The full-length *PRL1* cDNA was amplified by RT-PCR and ligated to pMAL-c5x (NEB) to generate MBP-PRL1. To generate the cCFP-PRL1 fusion vector, the *PRL1* cDNA was first cloned into the pSAT4-cCFP-C vector [35]. The DNA fragment containing cCFP-PRL1 was released by I-SecI restriction enzyme and subsequently cloned into the pPZP-RCS2-ocs-bar vector. All the primers are listed in Table S1.

### Co-IP assay

In the PRL1-PoII co-IP experiment, proteins were extracted from the transgenic plants harboring the *pPRL1::PRL1-YFP* transgene and incubated with anti-YFP (Clontech) or anti-RPB2 antibodies coupled to protein G agarose beads (Clontech) for 4 h at 4°C. After the beads were washed five times with protein extraction buffer, proteins were resolved by SDS/PAGE. Anti-YFP and anti-RPB2 antibodies were then used to detect PRL1-YFP and RPB2, respectively. To test the interaction of PRL1 with components of the DCL1 complex, PRL1-YFP (YFP) was co-expressed with DCL1-MYC or SE-MYC in *N. benthamiana*. Protein extracts were then incubated with anti-YFP or anti-MYC antibodies coupled to protein G agarose beads. Anti-YFP and anti-MYC (MBL) antibodies were used to detect PRL1-YFP/YFP and DCL1-MYC/SE-MYC, respectively.

### Dicer activity assay

*In vitro* dicer activity assay was performed according to Qi et al and Ren et al [13], [31]. *MIR162b* and *pre-miR162b* RNAs were produced by *in vitro* transcription under the presence of [α-^32^P] UTP. In the dicer activity assay, protein extractions were incubated with [^32^P] labeled *MIR162b* or *pre-miR162b* in reaction buffer containing 100 mM NaCl, 1 mM ATP, 0.2 mM GTP, 1.2 mM MgCl_2_, 25 mM creatine phosphate, 30 µg/ml creatine kinase and 4 U RNase inhibitor at 25°C. After the reactions were stopped at 40, 80 or 120 mins, respectively, RNAs were extracted and resolved on PAGE gel. Radioactive signals were detected with a PhosphorImager and quantified by ImageQuant version 5.2.

### BiFC assay

Paired cCFP and nVenus constructs were co-infiltrated in *N. benthamiana* leaves for 40 h. YFP signals were then detected with a confocal microscopy (Fluoview 500 workstation; Olympus) at 488 nm with a narrow barrier (505–525 nm, BA505–525; Olympus).

### RNA analysis

Northern blot was used to detect small RNA abundance as described [13]. qRT- PCR was performed to detect the levels of pri-miRNAs, transcripts of miRNA targets and *GUS* using cDNA templates reverse transcribed by the SuperScript III (Invitrogen) and oligo dT18 primer. qRT-PCR was run on an iCycler apparatus (Bio-Rad). RNA pull-down were performed according to Ren et al [13]. *MBP* and *MBP-PRL1* were expressed in *E.coli*. *MIR162b*, *pre-miR162b*, dsRNAs and *ssRNA* were produced by *in vitro* transcription with T7 RNA polymerase at the presence [α-^32^P] UTP whereas DNA was synthesized at IDT and labeled with T4 PNK at the presence [γ-^32^P] ATP. [^32^P]-labeled probes are incubated with amylose resin beads combined MBP or MBP-PRL1 at 4°C for 1 hour. After 4 times wash with washing buffer, DNA or RNA are extracted and resolved on PAGE gel. Radioactive signals were detected with a PhosphorImager and quantified by ImageQuant version 5.2. RIP was performed according to [13], [36]. Seedlings of transgenic plants harboring the *pPRL1::PRL1-YFP* transgene or *YFP* were used to examine the RNA binding activity of PRL1 *in vivo*. All the primers are listed in Table S1.

RNA half-life assay was performed according to Lidder et al [30]. Two-week-old Col and *prl1-2* seedlings were transferred to flask with incubation buffer (1/2 MS medium), respectively. After 30 min incubation, 3′-deoxyadenosine (Cordycepin, Sigma) was added to final concentration of 0.6 mM (time 0). Seedlings were collected at various time points (0, 15, 30, 60, 90, 120 and 240 min). qRT- PCR then was performed to detect the transcript levels of pri-miRNAs and *DDL*. For quantification, the transcript levels of pri-miRNAs and DDL at various time points were normalized to that of eIF4a, respectively. Value of time 0 was set to 1. Error bars indicate standard deviation of three technical replications. Three biological repeats were performed and similar results were obtained.

### Gel filtration analysis

The gel filtration was performed on an HPLC system and a HiPrep 16/60 Sephacryl S-300 HR column (GE Healthcare) at a rate of 0.5 ml/min, and 0.5 ml fractions were collected every minute. Fractions were separated by 8% SDS–PAGE and analyzed by Western blotting using antibodies recognizing CDC or YFP. The protein standards (Bio-Rad, http://www.bio-rad.com/) were used to calibrate the column contain five size standards.

## Supporting Information

Figure S1 (A) The expression of *MAC3b*, *MOS4*, *PRL1* and *SNC1* in four null mutants detected by RT-PCR. The T-DNA line of *mac3b* (SALK_050811), *mos4* (SALK_090851C), *prl1-2* (SALK_039427), *snc1* (SALK_047058C) are all in Columbia-0 genetic background. (B) The transcript levels of several small RNA targets in *prl1-2*, Col and complementation line. The amount of target transcripts in *prl1-2* and complementation line were normalized with *UBQUITIN5* (*UBQ5*) and compared with that of Col (set as 1). Error bars indicate standard deviations of three technical replications. *:P<0.05; **:P<0.01.
(TIF)

Figure S2 (A) The levels of *GUS* mRNA l in *PRL1^+^* and *prl1-2* harboring *pMIR172a*::GUS. *GUS* mRNA levels were determined by qRT-PCR and normalized to *UBQ5*. Value of *PRL1^+^* was set to 1. Standard deviation of three technical replications was shown as error bars. (B) *Pri-miR164a*, *pri-miR167a* and *DDL* mRNA decay in the half-life assay. Two-week-old Col and *prl1-2* seedlings were treated with 0.6 mM 3′-deoxyadenosine (Cordycepin, Sigma) at various times (0, 15, 30, 60, 90, 120 and 240 min). qRT-PCR was performed to detect pri-miRNA, and *DDL* transcription levels and normalized to internal control (*eIF4a*). Value of time 0 was set to 1. Error bars indicate standard deviation of three technical replications.
(TIF)

Figure S3 The PRL1-YFP transgene restores *in vitro* processing of *MIR162b* and *pre-miR162b* in *prl1-2*. Protein extracts isolated from inflorescences of Col, *prl1-2* and prl1-2 containing a PRL1-YFP transgene were incubated with *MIR162b* and *pre-miR162b* for 120 min.
(TIF)

Figure S4 The effects of *prl1-2* on the expression of several genes involved in miRNA biogenesis. (A) The transcript levels of several genes involved in miRNA biogenesis determined by qRT-PCR. *UBQ5* was used as a control. Standard deviation of three technical replications was shown as error bar. (B) DCL1, (C) HYL1 and (D) CDC5 protein levels in various genotypes detected by western blot. Controls were *dcl1-9* containing a truncated DCL1 protein, *hyl1-2* lacking of HYL1 and *cdc5-1* lacking CDC5.
(TIF)

Figure S5 Gel filtration analysis of CDC5 and DCL1. Col and *prl1-2* protein extracts from inflorescences were separated by HPLC. Eluted fractions were separated by SDS–PAGE and detected by Western blotting using anti-CDC5 or anti-DCL1 antibodies. Elution times of protein standards are shown on the top of the blots.
(TIF)

Table S1 Primers used in this study.
(DOC)
