# Supplementary figures and images for: PRL1, an RNA-Binding Protein, Positively Regulates the Accumulation of miRNAs and siRNAs in Arabidopsis
Source: PLoS Genet. 2014 Dec 4;10(12):e1004841. doi: 10.1371/journal.pgen.1004841 (PMC4256206; doi:10.1371/journal.pgen.1004841)

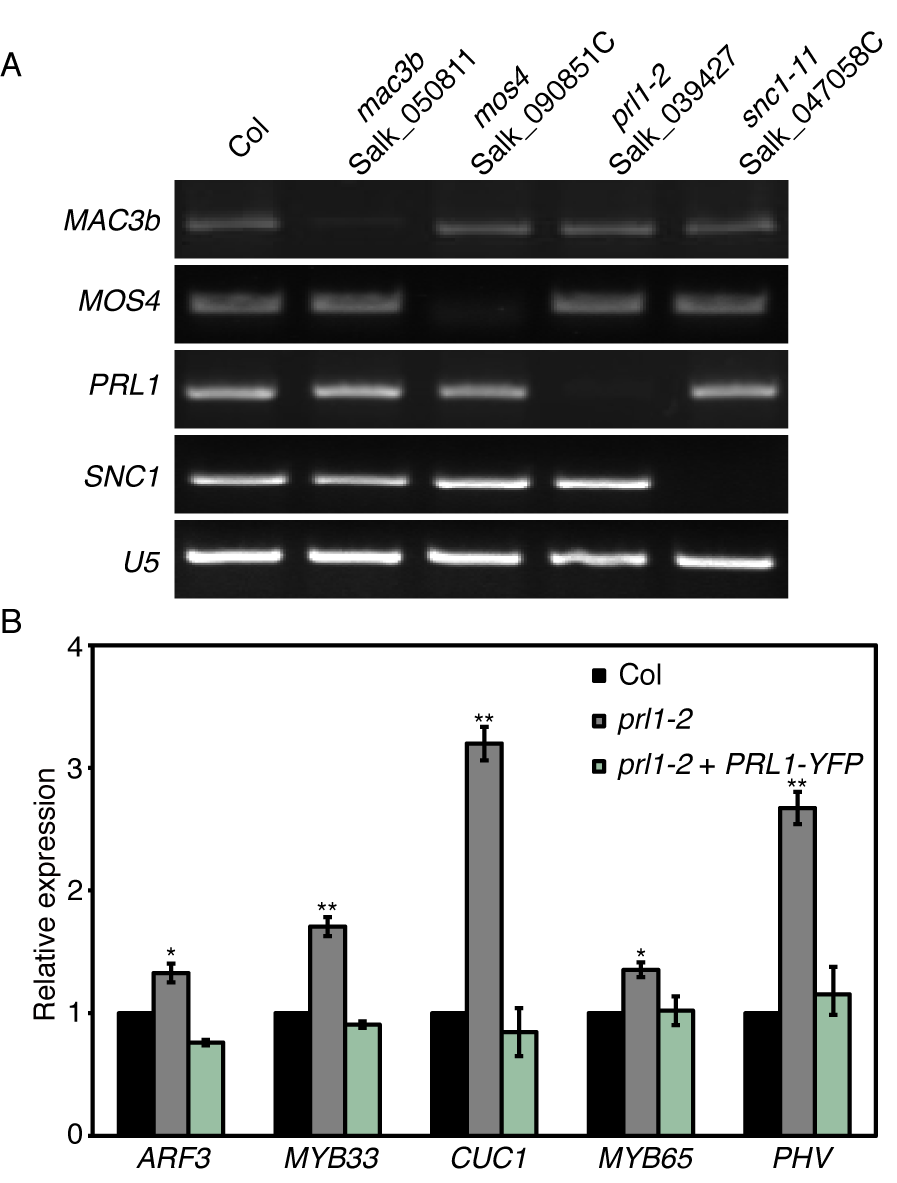

Supplement: Figure S1 — (A) The expression of MAC3b, MOS4, PRL1 and SNC1 in four null mutants detected by RT-PCR. The T-DNA line of mac3b (SALK_050811), mos4 (SALK_090851C), prl1-2 (SALK_039427), snc1 (SALK_047058C) are all in Columbia-0 genetic background. (B) The transcript levels of several small RNA targets in prl1-2, Col and complementation line. The amount of target transcripts in prl1-2 and complementation line were normalized with UBQUITIN5 (UBQ5) and compared with that of Col (set as 1). Error bars indicate standard deviations of three technical replications. *:P<0.05; **:P<0.01. (TIF) [file pgen.1004841.s001.tif]

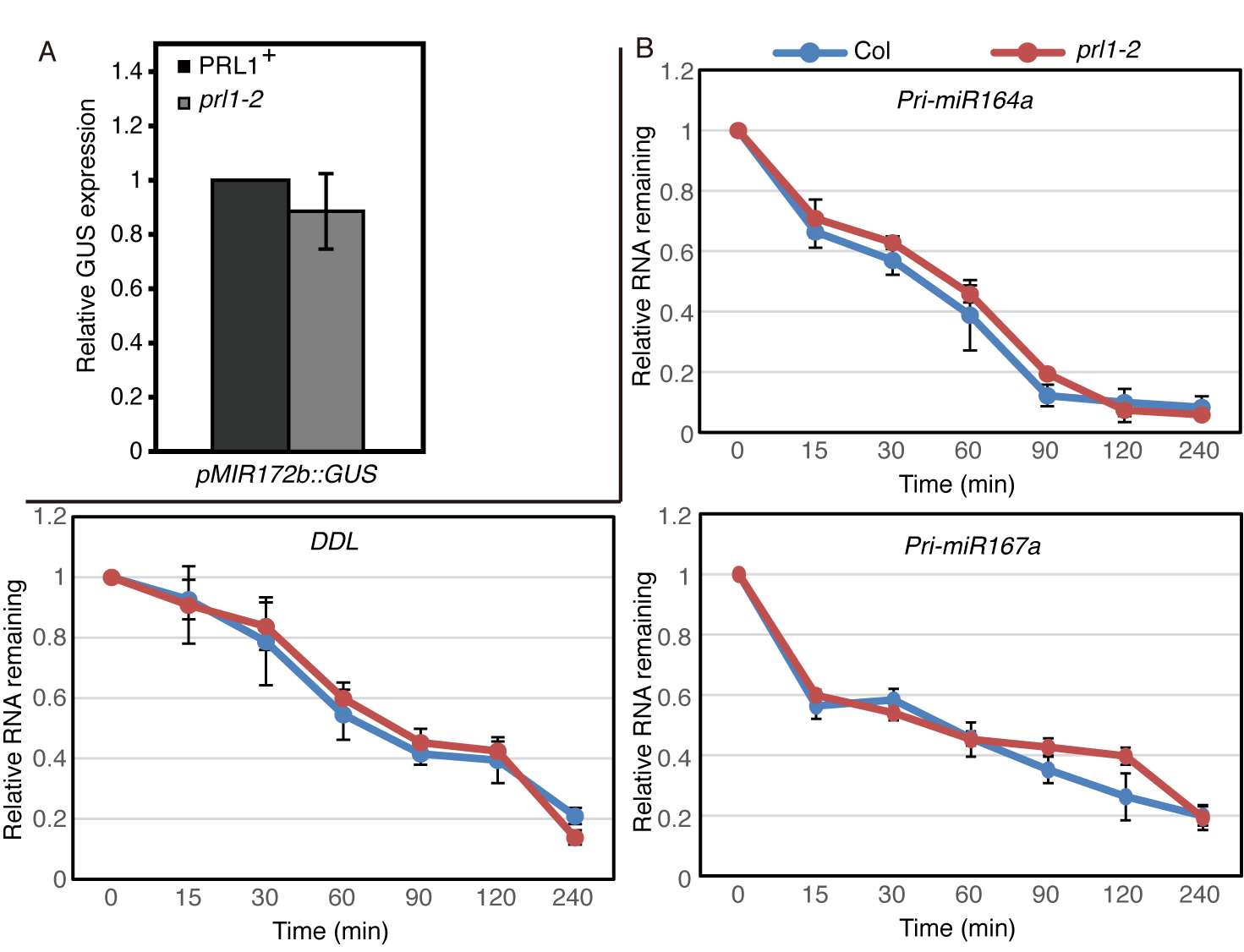

Supplement: Figure S2 — (A) The levels of GUS mRNA l in PRL1+ and prl1-2 harboring pMIR172a::GUS. GUS mRNA levels were determined by qRT-PCR and normalized to UBQ5. Value of PRL1+ was set to 1. Standard deviation of three technical replications was shown as error bars. (B) Pri-miR164a, pri-miR167a and DDL mRNA decay in the half-life assay. Two-week-old Col and prl1-2 seedlings were treated with 0.6 mM 3′-deoxyadenosine (Cordycepin, Sigma) at various times (0, 15, 30, 60, 90, 120 and 240 min). qRT-PCR was performed to detect pri-miRNA, and DDL transcription levels and normalized to internal control (eIF4a). Value of time 0 was set to 1. Error bars indicate standard deviation of three technical replications. (TIF) [file pgen.1004841.s002.tif]

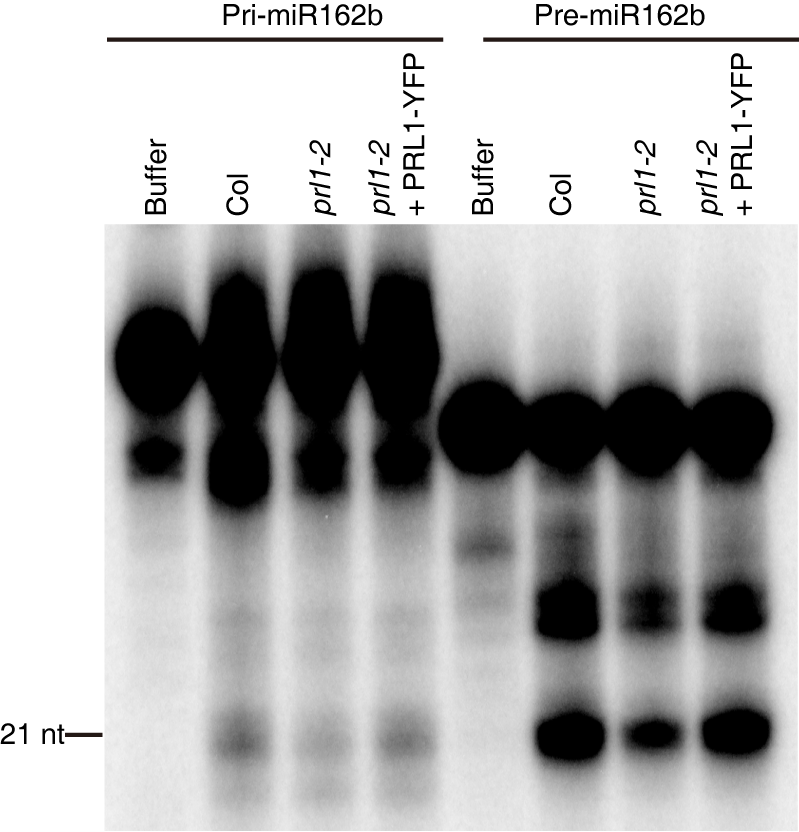

Supplement: Figure S3 — The PRL1-YFP transgene restores in vitro processing of MIR162b and pre-miR162b in prl1-2. Protein extracts isolated from inflorescences of Col, prl1-2 and prl1-2 containing a PRL1-YFP transgene were incubated with MIR162b and pre-miR162b for 120 min. (TIF) [file pgen.1004841.s003.tif]

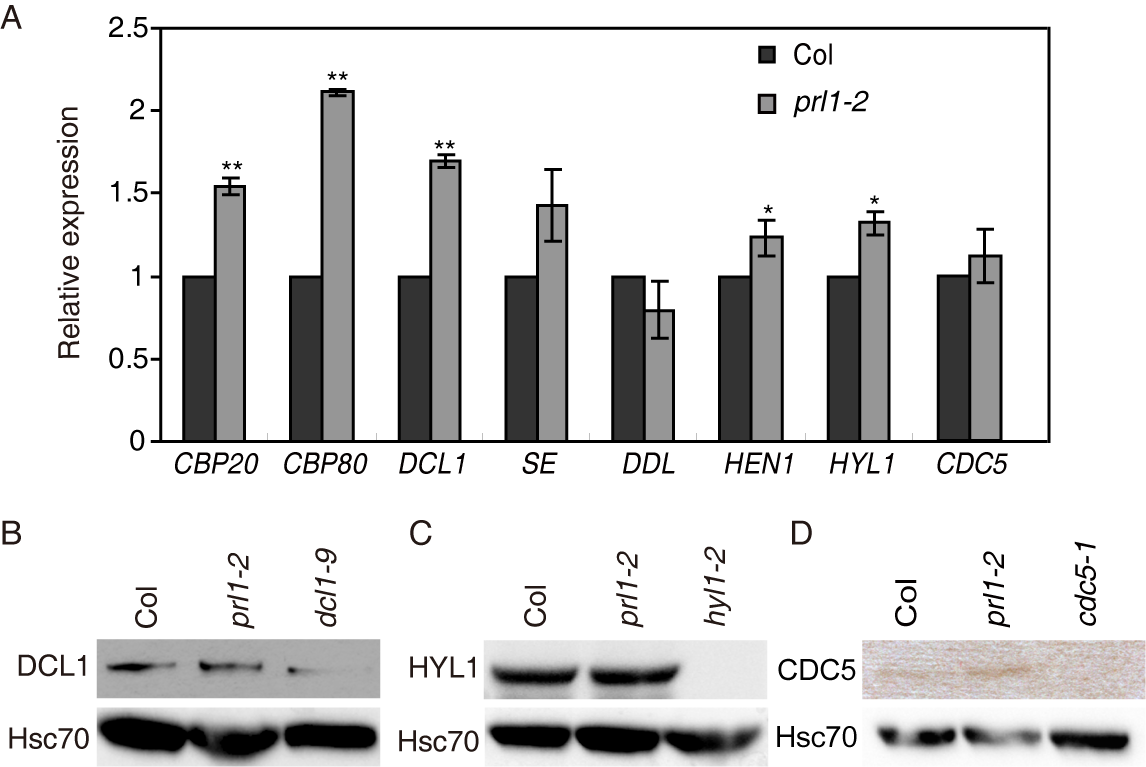

Supplement: Figure S4 — The effects of prl1-2 on the expression of several genes involved in miRNA biogenesis. (A) The transcript levels of several genes involved in miRNA biogenesis determined by qRT-PCR. UBQ5 was used as a control. Standard deviation of three technical replications was shown as error bar. (B) DCL1, (C) HYL1 and (D) CDC5 protein levels in various genotypes detected by western blot. Controls were dcl1-9 containing a truncated DCL1 protein, hyl1-2 lacking of HYL1 and cdc5-1 lacking CDC5. (TIF) [file pgen.1004841.s004.tif]

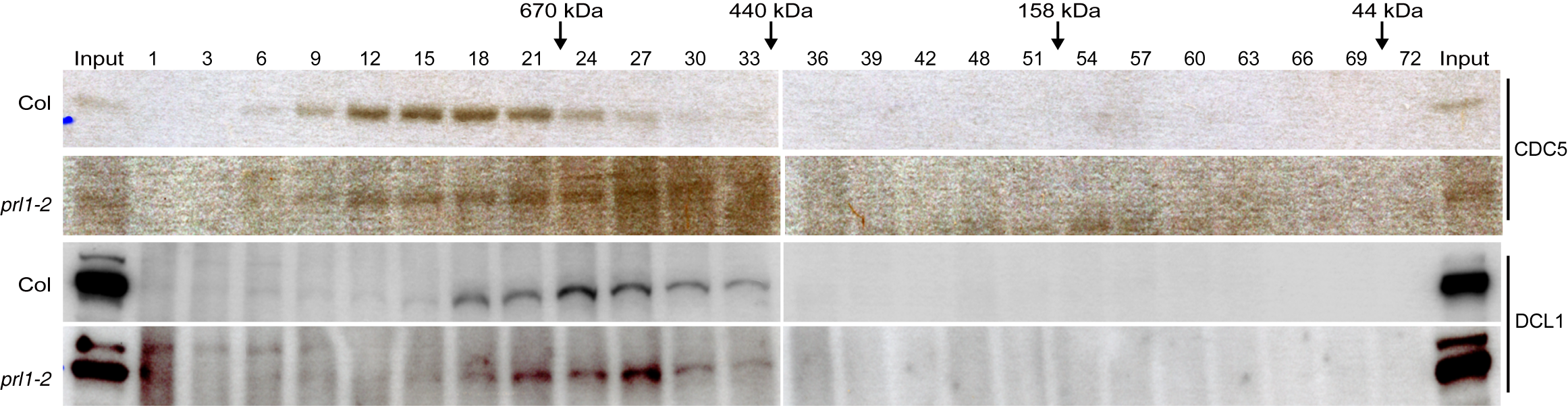

Supplement: Figure S5 — Gel filtration analysis of CDC5 and DCL1. Col and prl1-2 protein extracts from inflorescences were separated by HPLC. Eluted fractions were separated by SDS–PAGE and detected by Western blotting using anti-CDC5 or anti-DCL1 antibodies. Elution times of protein standards are shown on the top of the blots. (TIF) [file pgen.1004841.s005.tif]
