# Supplementary material for: PRL1, an RNA-Binding Protein, Positively Regulates the Accumulation of miRNAs and siRNAs in Arabidopsis
Source: PLoS Genet. 2014 Dec 4;10(12):e1004841. doi: 10.1371/journal.pgen.1004841 (PMC4256206; doi:10.1371/journal.pgen.1004841)
Supplement: Table S1 — Primers used in this study. (DOC) [file pgen.1004841.s006.doc]

| Name | Sequence (5’-3’) | Applications |
| --- | --- | --- |
| Primers for RT-PCR, RIP |  |  |
| GUS-F | CGATGTCACTCCGTATGTTATTG | qRT-PCR |
| GUS-R | CAGTTCTTTCGGCTTGTTGC |  |
| MIR159a-F | TCAGGAGCTTTAACTTGCCCTTT | RT-PCR/ RIP |
| MIR159a-R | CACGCTAAACATTGCTTCGGAAT |  |
| MIR 167a-F | cgacccttaaactctccataa | RT-PCR/ RIP |
| MIR 167a-R | ACTTCACCGTAGCAGATCAA |  |
| MIR 171a-F | tgctttggtagtagatgaggtt | RT-PCR/ RIP |
| MIR 171a-R | CGTGTGTGGTCAGGTAAGAT |  |
| MIR172a-F | ATCTGTTGATGGACGGTGGT | RT-PCR/ RIP |
| MIR172a-R | AATAGTCGTTGATTGCCGATG |  |
| Primers for constructs |  |  |
| PRL1cds-4F(KpnI) | GGGGTACCATGCCGGCTCCGACGACG | cCFP-PRL1 |
| PRL1cds-4R(KpnI) | GGGGTACCTTAGAAGCGCCTAATCTCCTTTGGTGG |  |
| PRL1c-F5 (SalI) | GCGTCGACATGCCGGCTCCGACGACGG | pMAL-MBP-PRL1 |
| PRL1c-R5 (EcoRI) | GCGAATTCTTAGAAGCGCCTAATCTCCTTTGGT |  |
| PRL1g-F | CACCTATTTATGTCTTAAACGACTGCC | pMDC204-PRL1-YFP |
| PRL1g-R | GAAGCGCCTAATCTCCTTTGGTGGT |  |
